# Mexican contributions to the electrophysiology field

**DOI:** 10.1016/j.hroo.2023.11.005

**Published:** 2024-03-20

**Authors:** Pedro Iturralde-Torres, Alejandra Iturralde-Chavez

**Affiliations:** ∗ Department of Diagnosis and Treatment, National Institute of Cardiology Ignacio Chávez, Mexico City, Mexico; † Pediatric Cardiology Research Department, Texas Children’s Hospital, Baylor College of Medicine, Houston Texas

**Keywords:** Electrophysiology/Mexican, Atrial flutter/reentrance, Heart conduction system/physiology, His bundle/right bundle branch block/Ebstein anomaly, Wolff-Parkinson-White pathways/algorithm, Pacemaker/reuse

## Overview

Although often perceived as a modern discipline, the foundations of electrophysiology can be traced back to Luigi Galvani’s descriptions of bioelectricity in animals in 1791.^1^ However, it was not until 1901 that Willem Einthoven recorded the first human electrogram, cementing his reputation as the pioneer of electrophysiology.^1^^,^^2^ Numerous groundbreaking discoveries have since shaped this subspecialty.

In this review, we describe and summarize the significant original contributions from Mexico to the electrophysiology field over the past 8 decades. We highlight the pivotal roles played by both past and present Mexican researchers in advancing our understanding of the electrical system, arrhythmias, and their treatments.

## Introduction

The history of clinical electrophysiology is long; however, the identification and treatment of cardiac arrhythmias first occurred in this century.^3^^,^^4^ While ancient Chinese and Arabic cultures have long used pulse measurements as diagnostic tools, it was not until the 19th century that the field of physiology truly blossomed, propelled by a slew of innovative instruments that revolutionized research.^3^ Willem Einthoven, embarked on this journey with the Lippamann capillary electrometer in 1895.^1^ By 1901, with the assistance of a massive 600-lb machine and a team of 5 operators, he successfully recorded the first human electrogram.^1^ This monumental achievement earned him the Nobel Prize in Medicine in 1924, recognizing his groundbreaking work in unveiling the mechanisms of the electrocardiogram (ECG).^1^^,^^2^

In this review, we endeavor to spotlight the most important and original contributions from Mexico to the world of electrophysiology. This is our tribute to the dedicated researchers who have wholeheartedly committed their lives to unraveling the intricacies of cardiac rhythm pathophysiology.

## Contribution to the studies on atrial flutter

Initially, in 1946 Weiner and Rosenblueth^5^ wrote the first paper presenting a case series that showed the type of electrical propagation in atrial flutter (AFL). The “circus movement” theory proposed by that time that AFL arose from 1 or more impulses perpetually propagating in a unidirectional loop around a specific closed path. Therefore, Weiner and Rosenblueth described that for this circuit 1 or more obstacles were needed as well as those obstacles being surrounded by conducting tissue. Moreover, the obstacle should have an effective perimeter, long enough to contain the wave represented by the impulse, in order that the front of this wave may always find nonrefractory tissue ahead.^5^ By 1947, Rosenblueth and Garcia-Ramos^6^ started studying the influence of artificial obstacles on experimental atrial flutter. After initially injuring the intravenous region in the right atrium, they methodically introduced a succession of additional injuries. Beginning at the lower periphery of the inferior vena cava’s orifice, these injuries progressively extended toward the atrioventricular (AV) groove. Once the injuries reached the atrium’s boundary, the flutter ceased and remained noninducible, effectively signifying the blockade of the cavotricuspid isthmus. Little did they realize that they had inadvertently described the inaugural ablation of the cavotricuspid isthmus.^6^

## Electrical activity of the bundle of His

In 1958, Alanís et al^7^ aimed to study an explanation for the on time interval elapsing between the activation of the atrium and the ventricle. Considering the fact that the AV node is a structure where the impulse coming from the atrium traverses on its way toward the ventricle, it seemed likely that an explanation for the AV latency could be found by studying its activity. By that time, little was known about the functional properties of the bundle of His. Alanis et al conducted experiments in isolated perfused hearts of dogs and cats. They introduced the exploring electrodes into the region where the node and the bundle of His were known to be located, and a small action potential was recorded. The temporal relationships and characteristics of this potential (His potential) suggested that it belonged neither to the atrium nor to the ventricular electrogram. Notably, the His potential evaded detection when electrodes were positioned in proximal or distal zones or adjacent to the anterior periphery of the coronary sinus orifice. However, consistent traces of the His potential were observed when electrodes adhered to the bundle’s trajectory. This evidence justified the hypothesis that the His potential originates in the bundle of His. The amplitude of the His potential does not changed along the course of the bundle of His.^7^

The culmination of this research furnished compelling evidence that the registered action potential was not an artifact of the AV node. Rather, it was emblematic of the activity of the bundle of His, as depicted by the His electrogram, and does not require the previous activation of the atrium. The His potential appeared between the auricular and ventricular electrograms. This conclusion justifies the subdivision of the AV interval into the subintervals AH and HV. Finally, since the introduction of an exploring electrode into the region where the bundle of His is located does not produce any block while the introduction into the region of the AV node did so, the activation of the bundle of His may be attributed to the activity previously originated in the AV node.^7^

## Electrogram of the conductive tissue in a normal canine heart

The Mexican electrocardiography witnessed a transformative era, thanks to the endeavor of Demetrio Sodi Pallares, a disciple of F.N. Wilson from Ann Arbor, MI. Sodi Pallares founded the Mexican School of Electrovectorcardiography, a vanguard institution that commanded international acclaim for many decades.^8^ One of his biggest contributions was the study of the electrograms of the conductive tissue.^9^ In this paper, in 1959 they presented the tracing, which they believed had been obtained for the first time, corresponding to the electrical activity of the right and left branches of the bundle of His. This pioneering effort successfully documented simultaneous electrical activities across diverse segments of the conductive tissue, encompassing the AV node, bundle, branches, and Purkinje fibers.^9^

Michael Mirowski arrived as a research fellow to the National Institute of Cardiology Ignacio Chávez, Mexico City. From 1959 to 1961, Mirowski, under the mentorship of both Sodi Pallares and Enrique Cabrera, immersed himself in the intricacies of electrocardiography. Mirowski used to say that Sodi Pallares challenged him more intellectually but that Enrique Cabrera was the only genius he had ever encountered. During his stay in Mexico, Mirowski authored 4 seminal papers, delving into the vectocardiographic and hemodynamic correlations in both atrial and ventricular septal defects.^10^^,^^11^

## New ECG algorithm for the location of the accessory pathways using only the QRS complex polarity

In 1996, Iturralde et al^12^ proposed a new algorithm designed to pinpoint the location of accessory AV pathways using just a 12-lead ECG. Their analysis centered on the QRS complex polarity in leads DIII, V_1_, and V_2_, drawing data from 102 patients diagnosed with Wolff-Parkinson-White (WPW) syndrome, who displayed manifest preexcitation and subsequently underwent successful radiofrequency catheter ablation.

Their findings categorized accessory pathways on the heart’s right side into 3 distinct regions encircling the tricuspid annulus, while the left-sided pathways were delineated into 2 regions surrounding the mitral valve annulus.

Intriguingly, a predominant characteristic observed in 42 of 46 patients (91%) with left posterolateral accessory pathways was a positive QRS complex in leads III and V_1_. This observation demonstrated a sensitivity of 91% and a specificity of 95%. Similarly, 16 of 19 patients (84%) with left inferior paraseptal or inferior accessory pathways exhibited a negative QRS complex in lead III and a positive one in lead V_1_, yielding a sensitivity of 84% and a specificity of 98%.

In contrast, from the 37 patients with right sided accessory pathways, all 6 patients with right anterosuperior paraseptal accessory pathways consistently had a positive QRS complex in lead III, contrasted by a negative one in lead V_1_, resulting in a perfect sensitivity of 100% and a specificity of 97%. In addition, of the 25 patients with right inferior paraseptal or inferior accessory pathways, 21 (84%) evidenced a negative or isodiphasic QRS complex in leads III and V_1_, yet presented a positive QRS complex in lead V_2_, registering a sensitivity of 84% and an unparalleled specificity of 100%. Finally, 5 of 6 patients (83%) with right anterior accessory pathways were characterized by a negative QRS complex across leads III, V_1_, and V_2_, revealing a sensitivity of 83% and a specificity of 96%.

In culmination, the application of this novel algorithm identified the location of the accessory pathways in 90 of the 102 patients studied, translating to an 88% success rate.^12^

## *SCN4B*-encoded sodium channel β4 subunit in congenital long QT syndrome

Congenital long QT syndrome (LQTS) is caused predominantly by mutations in genes that encode cardiac ion channels. In the intricate architecture of voltage-gated sodium channels, a central pore-forming α subunit is often accompanied by 1 or more supplementary β subunits. While 4 distinct β subunits are identifiable in cardiac tissue, none had previously been associated with any inheritable arrhythmia syndrome.

In 2007, Medeiros-Domingo et al^13^ presented a case of a 21-month-old Mexican mestizo girl with intermittent 2:1 AV block and an alarming prolonged corrected QT interval of 712 ms. Comprehensive genetic scrutiny of the 9 recognized LQTS-linked genes yielded no answers. However, a deeper dive into the Navβ subunits unearthed an L179F (C535T) missense mutation in *SCN4B* that cosegregated properly throughout a 3-generation family tree and was absent in 800 reference alleles. After this discovery, *SCN4B* was analyzed in 262 patients with genotype-negative LQTS (96% white) but no further mutations were found. Determined to understand the implications of L179F, the team used site-directed mutagenesis, replicating this mutation in HEK293 cells already expressing the *SCN5A*-encoded sodium channel α subunit (hNav1.5). In contrast to the wild type, L179F amplified the late sodium current 8-fold (when juxtaposed with *SCN5A* alone) and 3-fold (when compared with *SCN5A* + WT-β4). This surge in late sodium current mirrored the molecular and electrophysiological patterns typically observed with LQTS-related mutations.

They provide the first report to detail *SCN4B*-encoded Navβ4 as a novel LQTS-susceptibility gene (*LQT10*).^13^

## WPW syndrome in Ebstein anomaly

In 2007, the interplay between WPW syndrome and Ebstein anomaly came under rigorous scrutiny through a comprehensive review by Iturralde et al.^14^ The abnormal development of the tricuspid valve that occurs in these patients is described along with the mechanisms underlying conduction and rhythm disturbances including delayed intra-atrial conduction, right bundle branch block (RBBB), and ventricular preexcitation. They defined the ECG characteristics before and after ablation of an accessory AV pathway in patients with Ebstein anomaly. This research drew on a cohort from the National Institute of Cardiology Ignacio Chávez, encompassing 226 subjects. An observation was that 63 of these individuals (28%) exhibited documented tachycardia. Thirty-three patients with recurrent tachycardia were found to have a single right-sided accessory pathway that was successfully ablated. However, only 21 of 33 patients (62%) had a typical ECG pattern of preexcitation. In addition, none of the patients had an ECG pattern of RBBB during sinus rhythm. Yet, postablation, an overwhelming 94% exhibited the emergence of RBBB.^14^

The study unearthed a compelling insight: the lack of RBBB in patients diagnosed with Ebstein anomaly, who also suffered from recurrent tachycardia, was a potent indicator (with 98% sensitivity and 92% specificity) of the presence of an accessory pathway. Interestingly, one-third of the patients with Ebstein anomaly, who also experienced symptomatic tachyarrhythmias, showed either subtle or completely absent ECG indicators of ventricular preexcitation. The key takeaway was that, for such patients, the absence of an RBBB pattern strongly hinted at an underlying accessory pathway.^14^

## Association of congenital, diffuse electrical disease in children with normal heart: Sick sinus syndrome, intraventricular conduction block, and monomorphic ventricular tachycardia

In the present work, Iturralde-Torres et al^15^ reported 3 children with sick sinus syndrome, intraventricular conduction disease, sustained monomorphic ventricular tachycardia, and structurally normal heart. A defining trait shared among these children was the inducible, lidocaine-sensitive, monomorphic sustained ventricular tachycardia. One of the 3 even underwent successful radiofrequency catheter ablation.

Patients exemplified broad-spectrum and innate electrical dysfunction, affecting areas ranging from the atria, sinus node, AV node to the ventricular myocardium. Cardiac imaging studies did not reveal structural abnormalities in any case.^15^

The triad of sick sinus syndrome, intraventricular conduction impairment, and persistent monomorphic ventricular tachycardia emerged as the cardinal clinical and ECG manifestations. Such consistent presentations across patients suggest an underlying shared pathophysiological origin, casting these children as unique exemplars of congenital severe electrical derangements devoid of structural cardiac anomalies.^15^

## Low doses of quinidine for Brugada syndrome

The therapeutic potential of quinidine in mitigating Brugada syndrome has long been acknowledged. However, the limitation has often been the side effects accompanying the traditionally recommended dosages (1 g/d). In 2012, Márquez et al^16^ studied the efficacy of low doses of quinidine (≤600 mg/d) to prevent the recurrence of ventricular arrhythmias in Brugada syndrome. This research encompassed 6 male patients, each equipped with an implantable cardioverter-defibrillator. These individuals were primarily drawn from 2 institutions: National Institute of Cardiology and La *Pitié-Salpêtrière* Hospital in Paris, France. The initiation of quinidine treatment was typically postarrhythmic syncope or after the administration of appropriate shocks, with a particularly notable subset being the 4 patients who had experienced arrhythmic storms.

The findings were that quinidine, even at these reduced dosages, prevented recurrence of ventricular arrhythmias in all patients without side effects during a median follow-up of 4 years (ranging from 2 to 8 years). Three of the patients experienced recurrence of ventricular arrhythmias after cessation of the medication. In sum, Márquez et al^16^ underscored that these low quinidine dosages were not only well tolerated but also efficaciously prevented the resurgence of ventricular arrhythmias, encompassing arrhythmic storms, in patients with Brugada syndrome fitted with an implantable cardioverter-defibrillator.

## Reuse of pacemakers

The elevated cost of pacemaker implantation often poses a daunting barrier to patients in developing nations, delaying or even denying them access to this lifesaving intervention. One innovative solution to this challenge has been the reuse of pacemakers. In a 2013 study, Nava et al^17^ chronicled the experience at the National Institute of Cardiology Ignacio Chávez in Mexico City, where this practice was actively pursued.

Given the obvious concerns surrounding the safety and efficacy of reused pacemakers, Nava’s study rigorously compared outcomes between patients receiving reused devices and those implanted with brand-new ones. This ambispective noninferiority study spanned a decade, from 2000 to 2010, and encompassed a cohort of 603 patients. Of these, 307 were recipients of resterilized pacemakers while 296 were fitted with new devices.^17^

The researchers assessed 3 primary outcomes: unexpected battery depletion, postprocedural infections, and device malfunctions. In terms of individual outcomes, 1.7% of the new devices and 3.6% of the resterilized ones experienced unexpected battery depletion. Infections associated with the procedure were reported in 3.7% of patients with new pacemakers and 3.2% of those with reused devices. Notably, only a single malfunction was reported in the reused pacemaker group.^17^

Nava’s conclusions were unequivocal: the reuse of pacemakers is both feasible and safe. Moreover, he posited that for patients in developing countries grappling with bradyarrhythmias, pacemaker reuse could be a lifesaver. The only tangible downside to this approach is the anticipated shorter battery life span of reused devices. Yet, when stacked against new devices, reused pacemakers did not exhibit any significant inferiority.^17^

## Declaration of generative AI and AI-assisted technologies in the writing process

During the preparation of this work, the authors used ChatGPT in order to improve the quality of English writing, in a more proper and formal way. After using this tool/service, the authors reviewed and edited the content as needed and take full responsibility for the content of the publication.
